# Next generation of ALDH substrates and their potential to study maturational lineage biology in stem and progenitor cells

**DOI:** 10.1152/ajpgi.00420.2014

**Published:** 2015-02-05

**Authors:** Laurent Dollé, Luke Boulter, Isabelle A. Leclercq, Leo A. van Grunsven

**Affiliations:** ^1^Liver Cell Biology Lab, Vrije Universiteit Brussel (VUB), Brussels, Belgium;; ^2^MRC Human Genetics Unit, Institute for Genetics and Molecular Medicine, Edinburgh, United Kingdom; and; ^3^Laboratory of Hepato-Gastroenterology, Institut de Recherche Expérimentale et Clinique (IREC), Université catholique de Louvain (UCL), Brussels, Belgium

**Keywords:** AldeRed-588-A, Aldefluor, ALDH activity, liver progenitor cell

## Abstract

High aldehyde dehydrogenase (ALDH) activity is a feature of stem cells from normal and cancerous tissues and a reliable universal marker used to isolate them. There are numerous ALDH isoforms with preferred substrate specificity variably expressed depending on tissue, cell type, and organelle and cell status. On the other hand, a given substrate may be metabolized by several enzyme isoforms. Currently ALDH activity is evidenced by using Aldefluor, a fluorescent substrate likely to be metabolized by numerous ALDH isoforms. Therefore, isolation techniques based on ALDH activity detection select a heterogeneous population of stem or progenitor cells. Despite active research in the field, the precise role(s) of different ALDH isoforms in stem cells remains enigmatic. Understanding the metabolic role of different ALDH isoform in the control of stem cell phenotype and cell fate during development, tissue homeostasis, or repair, as well as carcinogenesis, should open perspectives to significant discoveries in tissue biology. In this perspective, novel ALDH substrates are being developed. Here we describe how new substrates could be instrumental for better isolation of cell population with stemness potential and for defining hierarchy of cell populations in tissue. Finally, we speculate on other potential applications.

stem cells (and their cancerous counterparts) are being extensively investigated for regenerative medicine and therapeutic targeting. They can be used as a direct cell source for regenerative medicine. They also provide experimental models for target discovery, toxicity testing, and drug screening. The ability to prepare pure, undamaged, and functional stem cells is a critical step for all such applications. Identification and isolation of these rarely occurring cell populations is challenging, and development of novel labeling approaches is needed to improve their detection and isolation, as an alternative to transgenic methods that preclude further utilization for regenerative medicine.

## Aldefluor Assay: One of the Main Driving Technologies To Identify Stem Cells

Increasing evidence suggests that aldehyde dehydrogenase (ALDH) activity is a reliable universal marker of stem cells and techniques based on presence and level of ALDH activity are used for sorting stem cells out of various normal and cancerous tissues [see reviews (2, 4, 9, 18, 22)]. ALDH is a family of enzymes with oxidoreductase activity implicated in many biological processes important for cell survival and cell protection such as lipid metabolism or detoxification of endogenous and exogenous hazardous aldehyde by-products (20, 28). ALDH activity is important for the development of epithelial homeostasis and, as a result, deregulation of this class of enzymes has been implicated in multiple cancers (2, 20). Aldehydes are widespread organic compounds found in the environment as well as endogenously formed during the metabolism of alcohols, amino acids, vitamins, retinoids, steroids and lipid peroxides, xenobiotics (e.g., acetaminophen, cyclophosphamide), and environmental toxics (e.g., cigarette smoke, motor vehicle fumes). Aldehydes are strong electrophilic compounds with terminal carbonyl groups that can form adducts with cellular components (proteins and nucleic acids), thereby initiating adverse biological effects, i.e., loss of protein activity and mutation of nucleic acids. Hence, disposal of aldehyde is a priority for cell protection and survival (28), even more so for long-living cells such as stem and progenitor cells. Hence, detection of high ALDH activity has been extensively used as a tool for stem cell isolation of different organs (2, 4, 9, 18, 22). Next to this application, our understanding of the biological function and consequences of ALDH activity in controlling stemness, activation, amplification, differentiation, and fate of this discrete cell population is limited.

The currently available commercial assay identifies ALDH^bright^ cells (cells with high ALDH activity) as those actively metabolizing Bodipy-amino-acetaldehyde, Aldefluor reagent (Aldagen; distributed by Stem Cell Technologies). This assay is highly sensitive, reproducible, nontoxic, and easy to use provided that you have access to a fluorescence-activated cell sorter (FACS). Aldefluor does not involve antibody recognition or the use of DNA-intercalating dyes and is also applicable to human material (8). Cytoplasmic enzyme activity detection has an advantage over antibody-based detection of cell membrane proteins because such activity is less likely to be damaged by enzymatic digestion and processing of the tissues, which are necessary steps for live single cell isolations. ALDH activity is detected by a fluorescent green emission, a technical limitation for cell sorting in organs (such as the liver) rich in endogenous fluorophores (flavins, NADPH) that autofluoresce in the green wavelength (∼480–580 nm). Green emission also reduces considerably the choice of combining fluorescently labeled antibodies to further fractionate the ALDH^bright^ population and preclude its use for cell isolation in tissues from green fluorescent protein (GFP) transgenic mice. An inevitable overlapping of green fluorescence emission into other channels is also problematic, and therefore “contamination” of ALDH expressing cells in other lineages can be high.

Numerous studies have documented the presence of ALDH^bright^ cells in several adult tissues and demonstrated that these cells carry significant stem/progenitor characteristics including stem cell markers, proliferative potential, sphere formation, clonogenicity, and multilineage differentiation. For example, sorting on ALDH activity enriches cell preparations for centroacinar and terminal ductal epithelial stem cells from mouse pancreas (25), for proximal airway basal (13) and prostatic stem cells (6), but also for hematopoietic (3, 11), breast (12), skeletal muscle (16), neural tissue (7), and colon (14) stem cells. Using this technique, we successfully identified an ALDH^bright^ population with features of hepatic stem/progenitor cells (HSPCs) in healthy mouse and human liver tissue (8). ALDH^bright^ putative HSPCs were estimated to comprise 2% of the nonparenchymal cell fraction in mouse livers and 35% of this sorted subpopulation exhibit stem cell features (K19^+^, EpCAM^+^, Sox9^+^, and CD29^+^) (8). Remarkably, about 5% cells in the low-ALDH-activity fraction (or ALDH^dim^) share stem cell markers as well, indicating that putative progenitors have escaped from detection by Aldefluor. It is currently unknown whether these cells express ALDH isoenzyme(s) with no or low affinity for the Aldefluor substrate, or with no or low overall ALDH activity, or represent a subpopulation of cells that have switched ALDH activity owing to, for instance, lineage specification or commitment. This illustrates perfectly the limitation of using a single ALDH substrate and green fluorescence emission.

## AldeRed-588-A: Another Alternative to Aldefluor for Detecting ALDH Activity

**Fig. 1.**
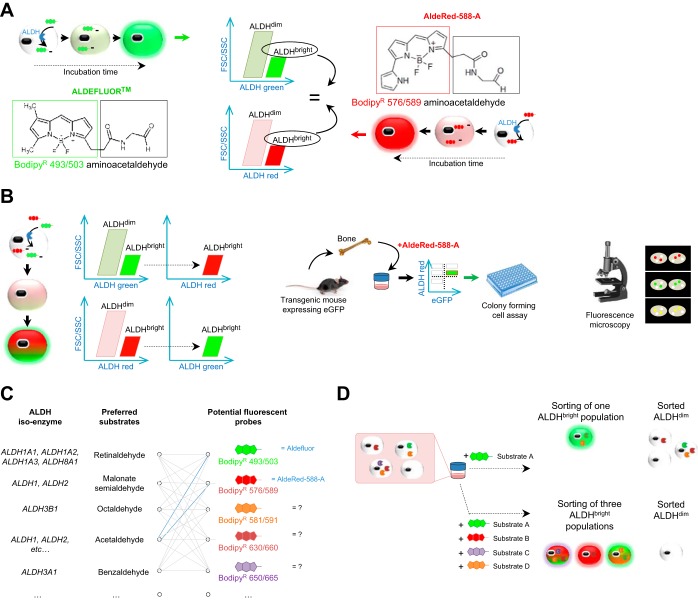
The AldeRed-588-A substrate provides a useful tool to select stem cells and opens up prospects for the next generation of aldehyde dehydrogenase (ALDH) substrates. *A*: Aldefluor and AldeRed-588-A have the same capacity to isolate an ALDH^bright^ cell population enriched in stem cells from a heterogeneous mixture of cells. *B*: AldeRed-588-A can be used for multicolor applications to fractionate ALDH^bright^ cells in the presence of green fluorophores, including the Aldefluor reagent and cells expressing enhanced green-fluorescent protein (eGFP) and can be visualized by fluorescent microscopy. *C*: emerging opportunities in generating preferred labeled substrates with different fluorescent probes. *D*: distinguishing distinct cell populations from a digested tissue on the basis of the specificity of the ALDH substrate might help in fractionating the different category of cells with ALDH activity referring to a certain physiological state. ALDH^bright^, cells with high ALDH activity; ALDH^dim^, low-ALDH-activity fraction; FSC, forward-scattered light; SSC, side-scattered light.

By their recent publication in *Nature Communications*, Minn and collaborators (21) rejuvenated the interest in using ALDH activity. They described a new red-shifted fluorescent ALDH substrate (AldeRed-588-A), for labeling of viable ALDH^bright^ cell populations. Structurally, Aldefluor (or Bodipy493/503-aminoacetaldehyde) and AldeRed-588-A (or Bodipy576/589-aminoacetaldehyde) have a common substrate moiety: acetaldehyde, indicating that a cell oxidizing Aldefluor is likely to have the same ability to metabolize the AldeRed-588-A substrate (Fig. 1*A*). In other words, AldeRed-588-A is considered as one of the other alternatives to Aldefluor substrate, without other functionally different characteristics. Elegantly, using cell lines known to express functional ALDH the authors demonstrated that Aldefluor and AldeRed-588-A essentially have the same efficacy and efficiency for identifying a ALDH^bright^ population (21). In addition, by successfully mixing two substrates, Minn and colleagues prove that labeling technique does not impede the structural recognition of the substrate by ALDH enzyme and that cell isolation of ALDH expressing cells is feasible by a single-step isolation method (Aldefluor and AldeRed-588-A are incubated simultaneously), thus avoiding additional purification or enrichment steps in which cells can be lost or damaged. This new AldeRed-588-A substrate has a broad emission spectrum, which currently precludes combination with a large array of fluorophores. Nevertheless, the technical innovation opens new avenues for stem cell research by offering a greater flexibility for ALDH^bright^ cell isolations.

Minn and coworkers (21) also proposed a protocol for using AldeRed-588-A with transgenic mice expressing enhanced-GFP, which is not an option with the Aldefluor green substrate because the emission spectra overlap. On the contrary, AldeRed-588-A can be used for multicolor applications to fractionate ALDH^bright^ cell populations in the presence of green fluorophores including the Aldefluor reagent itself or those cells expressing enhanced GFP (Fig. 1*B*). This is an important breakthrough in the field of stem cell biology, because the maturation and cell fate processes occurring during cell determination are often visualized by the tracking of the initial genetically labeled stem cells (by using fluorescent reporters such as GFP activated by Cre, for example) (5).

Beyond providing a useful tool to identify and isolate stem cells from different tissues, the authors show the real possibility of synthetizing functional substrates for ALDH enzymes. One could thus generate a library of fluorescently distinct substrates able to discriminate and fractionate stem cell populations by flow cytometry based on expression of specific ALDH isoenzymes (Fig. 1*C*). To act as a functional probe for ALDH, a compound should possess three important traits (21): an aldehyde moiety (“the substrate” recognized by the enzyme); a hydrophobic moiety for allowing free diffusion into any cells (supported by the Bodipy dye derivatives); and the capacity for further trapping within the cytoplasm (presumably by the different chemical groups linked to the Bodipy core) following conversion of the aldehyde into the corresponding carboxylic acid by ALDH. There are several possibilities in the choice of Bodipy dyes, and several studies have elucidated the importance of chemical groups on the Bodipy core to achieve and improve further functionality (e.g., water solubility, membrane permeability, narrowed wavelength absorption and fluorescence emission bands, compatibility with living cells, or long fluorescence lifetimes) (30, 31). The choice of the ALDH substrate, which would be linked to the Bodipy dye, will determine the binding affinity and specificity of the ALDH isoform (Fig. 1*C*). One substrate is likely to be metabolized by several ALDH isoforms and a given isoform is able to metabolize structurally diverse substrates. This infidelity makes it likely that any current ALDH assay is reflecting broad ALDH activity, with no discrimination between enzyme isoforms, and thus likely to identify different cellular subsets (17, 19, 20). Negativity of the assay may attest the absence of ALDH expression or, conversely, the expression of an enzyme toward which the tested substrate has no affinity. Indeed, ALDH activity has been related to different ALDH isoforms in different organs: ALDH1A1, ALDH2, ALDH3A1, and ALDH9A1 in hematopoietic tissue; ALDH1A1 and ALDH1A7 in pancreas (25); ALDH1/2 and ALDH3A1 in prostate (6); ALDH1A3, ALDH2, ALDH4A1, and ALDH5A1 in breast tissue (19); and ALDH1A1 in liver (8). Aldefluor and AldRed-588-A have been designed to detect a large array of ALDH enzymes and so a lack of isoform specificity is particularly problematic. One could envision differentially labeled ALDH isoform-specific substrates to create a library of probes to functionally discrete populations of cells expressing specific ALDH isoforms (Fig. 1, *C* and *D*). Such a strategy, already highlighted by Minn and coworkers (21), would help in fractionating different populations of cells and relate ALDH isoform expression to phenotype or physiological state but obviously needs further investigation and validation.

## Opportunities of Refining the Maturational Lineage Biology of Stem Cells and Their Progeny

A burning question in the stem cell field is whether progenitor cells need ALDH activity to fulfill their role or whether this activity is biologically inconsequential and only offers a convenient way to isolate a cell population with stem cell capacities. Obviously, ALDHs participate in many important biosynthetic processes implicated in cell protection from endogenous and exogenous aldehyde substrates (28, 29). However, the high expression levels of ALDHs in various tissues suggest that these enzymes have additional pivotal roles. Accumulating evidence suggests that the stem cell and/or tissue repair activity is influenced by the ALDH activity through production of retinoic acid, which has a potent biological activity (2, 9, 18, 20). Furthermore, ALDH activity is frequently required for cell proliferation, differentiation, and response to oxidative stress (4, 9, 19). Thus, besides acting as cell protectant, ALDH activity could be important for maintaining cellular integrity as well as regulating cell turnover and response. There are few studies exploring a role of ALDH activity in cell fate decision. One of the biggest reasons for this is the inability to trace the fate of stem cells concurrently with modulation of ALDH activity, to address whether these enzymes have a functional consequence for stemness and/or differentiation capacity.

In recent years work using tracing of precursor cell populations to adult fates has begun to shed light on the mechanisms and dynamics of stem/progenitor cell fate determination during development, tissue maintenance, and repair, as well as their dysregulation during carcinogenesis (5). Combining such tools with ALDH activity detection by using the next generation of substrates based on the AldeRed-588-A synthesis (21) could offer an important clue to relate the role ALDH activity in stem/progenitor cells biology, in their lineage organization, and/or in influencing the lineage restriction choices as they transit into progenitor cells and then mature cell types. In our previous studies, we were able to detect ALDH in the canals of Hering and in bile duct epithelial cells, the putative hepatic stem cell niche under normal, healthy physiological conditions (8). A distinct pattern of expression was observed following liver injury of any etiology: we found fast upregulation of the ALDH1A1 protein, followed by a return to regular expression once injury had resolved. This observation is compatible with the proposition that mature cells were generated from this ALDH^bright^ pool. Subsequently, we found that ALDH activity itself follows the same kinetics as the protein (Dollé L, van Grunsven LA, unpublished data), suggesting a potential relationship between ALDH activity and lineage commitment of hepatic stem/progenitors.

**Fig. 2.**
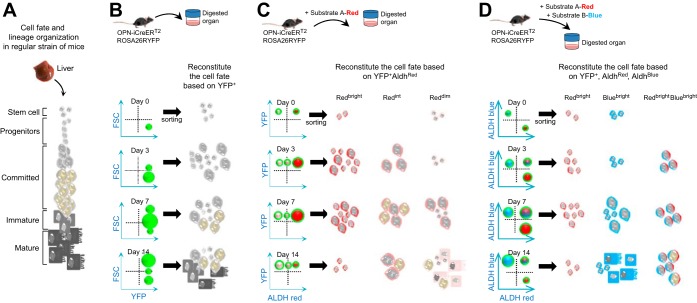
Opportunities in refining the metabolic hierarchy of stem cells and their progeny in the liver. *A*: general representation of the cell fate determination of the hepatic stem/progenitor cells (HSPCs) to the hepatocytic lineage and their organization is represented. *B*: to follow the fate of the stem cells and their progeny in the liver upon injury, OPN-iCreERT2;ROSA26RYFP mice could be used. All sorted cells are by definition green, and the green color is therefore not shown to avoid confusion. Reconstitution of the cell fate can be done by sorting yellow fluorescent protein (YFP)^+^ fractions at different time points after injury. *C*: hypothetically, combination of ALDH activity (here, red substrate) with genetic tracing allows an additional fractionation of the YFP^+^ population. A hypothetical scenario is given, which illustrates a potential bias in ALDH activity depending on cell fate. Red color has been added in cytoplasm for a simplistic view. *D*: as in *C*, but now using a mixture of 2 distinct fluorescent-labeled substrates (red and blue), which could even further refine the molecular features of the YFP^+^ sorted cells based on additional subfractionations. Red or blue colors in cytoplasm of cells represent the ALDH^bright^ populations. ALDH^int^ and ALDH^dim^ (for each substrate) are not represented to lighten the figure. With these additional parameters, it will definitively be possible to fractionate further the 3 ALDH^bright^ populations. ALDH^int^, intermediate aldehyde dehydrogenase activity; Aldh^Red^, red ALDH activity based on the use of Substrate A-Red; Aldh^Blue^, blue ALDH activity based on the use of Substrate B-Blue; Red^Bright^, high red ALDH activity; BlueBright: high blue ALDH activity; Red^Bright^Blue^Bright^, high red and high blue ALDH activities.

Figure 2*A* illustrates the general representation of the cell fate determination of the HSPCs to the hepatocytic lineage and their organization. Recently, OPN-iCreERT2;ROSA26RYFP mice have been used to determine the fate of these cells, which yielded functional hepatocytes in response to chronic liver injury (10). Additional genetic constructs have then been developed (15, 24, 26) illustrating the feasibility to trace the HSPC fate in adult tissues by use of multiple putative stem cell markers. The eventual reconstitution of the cell fate can be seen by sorting yellow fluorescent protein (YFP)^+^ fractions at different periods of injury, with no information about ALDH activity in these cells along their differentiation axis (Fig. 2*B*). Combination of ALDH activity (by using a red substrate) with YFP detection by flow cytometry on cells coming from OPN-iCreERT2;ROSA26RYFP mice, for example, would allow an additional fractionation of the YFP^+^ population based on ALDH copositivity. Indeed, the use of red substrate would enable one to determine which cells have ALDH activity (ALDH^bright^) vs. the ones without (ALDH^dim^); it would then be possible to better characterize which fraction (ALDH^bright^ or ALDH^dim^) correlates with a particular phenotype and to evaluate the potency of these subpopulations in vivo. This dynamic functional reconstitution allows then a better understanding of the regulators of cell fate compared with YFP^+^ cells alone (Fig. 2*C*). It is tempting to anticipate that the new generation of ALDH substrates will enable to discriminate cells expressing distinct ALDH isoform, including in the ALDH^dim^ population, and thereby provide tools to address the functional relevance of ALDH isoenzyme expression profiles for stem cell maintenance, lineage commitment, and cell fate. If indeed a shift in ALDH activity is instrumental in modifying cell behavior, then targeted modulation of the activity of this family of enzymes could be used for therapeutic purposes.

## Additional Benefits of Considering New ALDH Substrates

Exploiting the differences in isospecificity of new ALDH substrates may subsequently facilitate the design of new inhibitors selective for each isoform, which would allow, perhaps for the first time, modulation of the ALDH isoenzymes to favor a particular lineage outcome in HSPCs and other adult stem and progenitor cells. Additional benefits from identifying new ALDH substrates or inhibitors might greatly impact other fields, like cancer research and clinical diagnosis. ALDHs participate in multiple metabolic pathways and play a role in several cancerous disease states, including cancer chemoresistance, by metabolizing activated forms of oxazaphosphorine drugs. Among them, cyclophosphamide, mafosfamide, or analogs have been widely used for treatment of solid tumors but their efficacy is limited owing to resistance convened by overexpression or stabilization of ALDHs. Recognition, conception, and design of new ALDH inhibitors or competitive substrates could then be helpful in overcoming this escape mechanism to chemotherapeutic agents (23). Since early detection of cancer is key for curative treatment, there is considerable interest in noninvasive and inexpensive cancer diagnosis. Targeting ALDH as a potential approach for cancer cell labeling might be an appropriate “tracer” for discerning drug-sensitive and drug-resistant phenotypes of cancers to evaluate the effectiveness of novel therapies (27). Ultimately, targeted killing of a cell, based upon its specialized metabolic state (here ALDH activity), may be useful for clinical application to preferentially target those rapidly proliferating cells for growth inhibition or death, as has been described in the case of threonine dehydrogenase in mouse embryonic stem cells (1). In the field of cell therapy, tools for tracing transplanted cells are needed. Therefore, use of ALDH activity markers as imaging agents for assessing stem cell migration, engraftment, and expansion in vivo could greatly facilitate our understanding of the mechanisms of stem cell homing.

## Concluding Remarks and Future Directions

The understanding of the function of ALDHs in stem cell systems is underdeveloped, particularly given the broad range of cell types that have been isolated on the basis of ALDH activity by using the Aldefluor assay. Recent work by Minn et al. (21) not only describes the coupling of aminoacetaldehyde to a red fluophore (Bodipy 578/589) while maintaining the same specificity as the well-known Aldefluor substrate but also paves the way for the generation of a wide portfolio of ALDH substrates (coupled to different colors), which would facilitate functional studies of different ALDH isozymes in a variety of cellular settings. A better understanding of how ALDH isoenzymes mark (stem) cell populations of any organ will undoubtedly offer a robust and efficient approach for the isolation of these cells and their potential use in regenerative medicine (Fig. 2).

The generation of potentially new isoenzyme-specific ALDH substrates might lead to the discovery of new selective ALDH inhibitors, which will have far-reaching implications for tissue homeostasis and repair. Understanding the molecular mechanisms in which different ALDH isoforms actively play a role, in both normal and cancerous tissues, as well as the precise identification of specific ALDH isoforms prevalent in certain tumors, will have major diagnostic and prognostic implications.

## GRANTS

L. Dollé, L. A. van Grunsven, and I. A. Leclercq received funding from Interuniversity Attraction Poles (IAP), phase VII, contract P7/47 (Federal Science Policy; BELSPO). L. Dollé and L. A. van Grunsven received funding from Flemish Research grant (FWO) G.0348.13N. I. A. Leclercq received funding from PDR 7.106714 and is a senior research associate with the Belgian national fund for scientific research (FNRS). L. Boulter was funded by the Leverhulme Trust and the Medical Research Council.

## DISCLOSURES

No conflicts of interest, financial or otherwise, are declared by the author(s).

## AUTHOR CONTRIBUTIONS

L.D. conception and design of research; L.D. performed experiments; L.D. analyzed data; L.D. interpreted results of experiments; L.D. prepared figures; L.D. and L.B. drafted manuscript; L.D., L.B., I.A.L., and L.A.v.G. edited and revised manuscript; L.D., L.B., I.A.L., and L.A.v.G. approved final version of manuscript.
